# The behavioral study on the interactive aggravation between pruritus and depression

**DOI:** 10.1002/brb3.964

**Published:** 2018-05-01

**Authors:** Xiao‐Dong Wang, Gang Yang, Yang Bai, Yu‐Peng Feng, Hui Li

**Affiliations:** ^1^ Department of Anatomy and K.K. Leung Brain Research Centre The Fourth Military Medical University Xi'an China

**Keywords:** chloroquine, chronic unpredicted mild stimulation, depression, histamine, interactional aggravation, pruritus

## Abstract

**Background:**

The interactive aggravation of pruritus and depression is well‐known, but an appropriate experimental model that could mimic this behavioral phenomenon is still lacking. Thus, a systematic animal behavioral investigation was carried out in this study. This will promote the research and treatment of pruritus and depression.

**Methods:**

The 2,4‐dinitrofluorobenzene (DNFB)‐induced chronic itch model was established to measure the depression index by forced swimming test (FST), tail suspension test (TST), and splash test (ST). The chronic unpredicted mild stress (CUMS)‐induced depression model was established to measure spontaneous itch and acute histamine or chloroquine‐induced itch behaviors. A depression and itch combining model was also established to measure the scratching and depression behaviors. The motor function of DNFB mice was analyzed by the rotarod test.

**Results:**

The scratching number, the immobility time in the FST and TST, and the grooming number in the ST test were all significantly increased in the chronic itch model. Mice receiving CUMS treatment showed significantly increased spontaneous scratching number, immobility time in the FST and TST tests, and grooming number in the ST. The combined model showed increased immobility time in FST and TST tests and increased grooming number in ST comparing to the depression model, and showed increased scratching number comparing to the chronic itch model. After histamine (His) or chloroquine (CQ) injection, the scratching numbers of CUMS mice were all significantly increased compared to those of His‐ and CQ‐control, respectively. Anti‐depression drug ketamine could significantly inhibit the depression‐like behaviors of CUMS mice, and simultaneously stopped the promoting effect on His‐induced acute itch.

**Conclusions:**

This study established an appropriate cross aggravation experimental mode and demonstrated that there is cross aggravation between pruritus and depression. The illumination of related mechanisms underlying this cross aggravation effect will provide theoretical basis for the prevention and treatment of depression and pruritus.

## 1 INTRODUCTION

In clinical settings, chronic refractory pruritus afflicts patients both physically and mentally. Along the course of chronic pruritus, the vicious “itch‐scratch‐itch” cycle will impair the mental state, induce somnipathy, cognitive deficit, and anhedonia, eventually leading to depression (Lee, Stull, & Yosipovitch, 2017). In addition, some patients who suffer from long‐term intense social stress are diagnosed as “depression”. These ones usually exhibited the outbreak or the aggravation of dermatologic diseases, such as pruritus and neurodermatitis (Lee et al., 2017; Tey, Wallengren, & Yosipovitch, 2013). In light of these, chronic itch could lead to negative emotions. Conversely, negative emotions, such as depression, can also induce or aggravate itch (van Laarhoven et al., 2012), forming the vicious “itch‐depression‐itch” circle. Although people are familiar with this phenomenon, an ideal therapy for this kind of disease is still urgently needed in clinical practice. One reason accounting for this may be the mechanisms underlying pruritus and depression, especially the interactive aggravation between them, remain unclear.

The interactive aggravation of itch and depression can be observed in both clinic and real life, but there is no appropriate experimental behavioral model that could mimic this phenomenon. NC/Nga mice were originally established as an inbred strain in Japan. Under conventional conditions, NC/Nga mice frequently scratch their bodies by their hind paws, which leads to the development of dermatitis (Takahashi et al., 2005). Amano, Negishi, Akiyama, and Ishikawa (2008) observed that chronic stress (water avoidance stress, WAS) could induce atopic dermatitis‐like skin lesions even under specific pathogen‐free (SPF) conditions; however, mice without WAS treatment do not show spontaneous itch under SPF conditions. Zhao, Hiramoto, Asano, Kubo, and Sudo (2013) investigated the effect of WAS treatment on the number of scratching in mice given subcutaneous injection of compound 48/80 (a kind of pruritogen). They observed that the scratching number is increased in the WAS group compared with the sham group, and WAS mice exhibit increased number of mastocytes in the skin and increased level of serum histamine after compound 48/80 injection compared to the sham group. All of these results suggest that chronic stress, such as depression, could evoke or aggravate pruritus. Peng et al. (2015) found that chronic stress significantly enhances the scratching number when administrating 5‐HT into cheek skin in rats, and β_2_‐adrenoceptors may mediate itch hypersensitivity via upregulating proinflammatory factors, such as TNF‐α in the skin. All the studies mentioned previously concentrated on the facilitative effects of chronic stress (depression) on itch hypersensitivity instead of the effects of chronic itch on depression. Meanwhile, the type of depression model is not the most common mild stress model. Besides, no behavioral model exhibiting the interactive aggravation of depression and itch, which is urgently needed in preclinical investigations, has not been reported up to now.

In this study, we established DNFB 2,4‐dinitrofluorobenzene induced chronic itch model (Terakawa et al., 2008; Zhang et al., 2014) and CUMS (chronic unpredicted mild stress)‐induced depression model (Mao, Ip, Ko, Tsai, & Che, 2009), and conducted a series of depression and scratching behavior tests to establish the behavioral paradigm of interactive aggravation between pruritus and depression in mice, and thus identified the vicious cycle of “itch‐depression‐itch” (Mao et al., 2009; Willner, 2017). This study will pave the way for underlying mechanisms of this behavioral paradigm and provide theoretical basis for clinical treatment of psychogenic pruritus and pruritus‐related mental disorders.

## 2 MATERIALS AND METHODS

### 2.1 Experimental animals

A total number of 100 healthy male C57/BL6 mice (4‐week old, 16–19 g) from the Laboratory Animal Center of the Fourth Military Medical University (Xi'an, China) were used in this study. Food and water were available free. Mice were acclimated for 1 week before the experiments. The room was under controlled conditions of temperature (set point 22°C), air humidity (set point 50%) and a 12‐hr light/dark cycle (lights on at 7:00 a.m., lights off at 7:00 p.m.). All experimental procedures in this study were approved by the Committee of Animal Use for Research and Education in the Fourth Military Medical University.

### 2.2 Experimental design

Five sets of experiments were performed. In the first experiment, 20 mice were randomly and equally divided into DNFB‐induced chronic itch model (DNFB model) and control groups. We examined the depression behaviors of DNFB‐treated and control animals with the aim of investigating whether chronic itch could induce depression‐like behaviors via forced swimming test (FST), tail suspension test (TST), and splash test (ST). Meanwhile, the motor function of DNFB mice was analyzed by the rotarod test. In the second experiment, 20 mice were randomly and equally divided into chronic unpredicted mild stress (CMUS)‐induced depression model and control groups. Twenty‐eight days later, FST, TST, ST, and spontaneous itch test were proceeded. Histamine (10 mg/ml, Sigma) or chloroquine (10 mg/ml, Sigma)‐induced acute itch behavior was also observed in the CMUS model. In the third experiment, 30 mice were randomly and equally divided into three groups (A, B, and C). Group A received CMUS stimulation as well as chronic DNFB treatment for 14 days, while group B and C received DNFB and CMUS treatment for 14 days, respectively. Scratching number and depression index were compared among group A, B, and C. Finally, in the fourth experiment, 30 mice were randomly divided into three groups (A, B, and C), and each group consisted of normal control and treatment subgroups. Group A received intraperitoneal injection of ketamine (30 mg/kg, 0.2 ml) on the 0th and 14th CUMS‐treating day (Brachman et al., 2016); Group B received intraperitoneal injection of ketamine as well as CUMS treatment; Group C received intraperitoneal injection of 0.9% saline as well as CUMS treatment. The scratching number after histamine injection and the immobility time in the FST were measured 28 days after ketamine administration to test the hypothesis whether chronic depression promotes itch.

### 2.3 Experimental protocols

#### 2.3.1 Chronic itch model

In this study, DNFB‐induced chronic itch model was established by repeatedly applying DNFB to the skin which could cause atopic dermatitis‐like pathological alterations and vigorous scratching behaviors in mice and thus is considered an ideal chronic itch model (Terakawa et al., 2008; Zhang et al., 2014). Twenty male C57/BL6 mice were randomly and equally assigned to DNFB model and control groups. Splash test was carried out to all the mice before the treatment and no depression‐like behaviors were observed in these two groups. DNFB model mice were sensitized by applying 100 μl of 0.15% DNFB acetone (Sigma) solution on a ~2 cm^2^ area of fur‐shaved abdominal skin for initial sensitization. Seven days later, 50 μl of 0.15% DNFB acetone solution was applied twice a week (every 2–3 days) to the shaved nape of mouse for 5 weeks as challenge. During this treatment, the skin gradually crusted and fell off, and there were also many scratching marks. After 35 days, the back skin of neck was naked and hairless without scratching marks. To examine spontaneous itch, scratching was measured 24 hr after DNFB administration for 30 min. DNFB model was considered to be successful if the scratching number was greater than 150. Compared with DNFB model group, the control group was performed the same procedures except that the acetone solution was DNFB‐free.

#### 2.3.2 Chronic unpredicted mild stress model

Chronic unpredicted mild stress‐induced depression model, an ideal chronic depression model, was established by administrating a variety of stressful stimuli to mice for 4 weeks which successfully showed depression‐related behaviors (Mao et al., 2009). Twenty mice were randomly and equally divided into CUMS model and control groups. Splash test was carried out to all the mice before the treatment and there was no depression‐like behavior in these two groups. The following stressors were presented in a pseudo‐random order during each weekly cycle: overnight illumination (16 hr periods), clip tail suspension (1 min periods), food deprivation (24 hr periods), water deprivation (24 hr periods), constraint (6 hr periods), cage tilt (45°, 24 hr periods), swimming in ice water (5 min periods), soiled bedding (24 hr periods). The CUMS exposure period was 28 days (Tan et al., 2015). The control group was kept as usual. The CUMS‐induced depression model was assessed by the FST, TST, and ST tests.

#### 2.3.3 Chronic itch‐depression model

Thirty mice were randomly and equally assigned to three groups. Splash test was carried out to all the mice before the treatment and there was no depression‐like behavior in these three groups. Mice in group A were treated with both DNFB and CUMS for 14 days. Mice in group B and C were only treated with CUMS and DNFB separately for 14 days.

#### 2.3.4 Forced swimming test

The mice were individually placed in transparent and organic glass bucket (20 cm high, 14 cm in diameter) with a depth of 15 cm and water temperature of 25 ± 1°C. The time that the mice did not move in the water during 5 min period was observed and recorded. After each experiment, the inside walls of the bucket were rinsed and the water was changed in order to avoid any impacts on the next test (Mao et al., 2009).

#### 2.3.5 Tail suspension test

The tail of mice was hung to the holder upside down with the head of mice 15 cm from the table before a white background. The time of immobility in the last 5 min was recorded during a 6‐min time duration, and then analyzed via software (Smart 2.5) (Can et al., 2012).

#### 2.3.6 Splash test

The mice were individually placed in the cage which simulated the normal living environment, and adjusted to the environment 30 min before. And then, the 30% sucrose solution was splashed to the mice lightly with to damp the back of their neck for three times. The frequency of grooming was counted. In order to avoid any impacts on the next test, the cage was splashed with 75% ethyl alcohol and padding was changed (Pesarico et al., 2016).

#### 2.3.7 Rotarod test

The rotarod test was performed to assess the motor coordination of mice. Before test, mice were trained for three successive days at a constant speed (5 r/min) for 3 min. During the test, rats were placed on the rotarod apparatus (Shanghai Biowill Co. Ltd, Shanghai, China), with rotation starting at 0 r/min and progressing to a maximum of 30 r/min within 6 min. The falling latency of mice was recorded. Each mouse was measured three times (Shiotsuki et al., 2010; Zhang et al., 2015).

#### 2.3.8 Scratching behavior test

All scratching behavioral tests were videotaped by SONY HDR‐PJ410 digital video camcorders from a side angle. The videos were played back on a computer, and the quantification of mice behavior was performed by persons who were blind to the treatments (Sun & Chen, 2007; Sun et al., 2009).

### 2.4 Statistics

The IBM SPSS 13.0 and GraphPad Prism6.0 packages were used to analyze and plot the results. Before applying any statistics, the data were checked for normal distribution. Student's *t*‐test was used for statistical analysis. These data were presented as the mean ± standard error of the mean (SEM). The statistical significance was set at *p *<* *.05.

## 3 RESULT

### 3.1 The establishment of chronic itch model and its depressive behaviors

**Figure 1:**
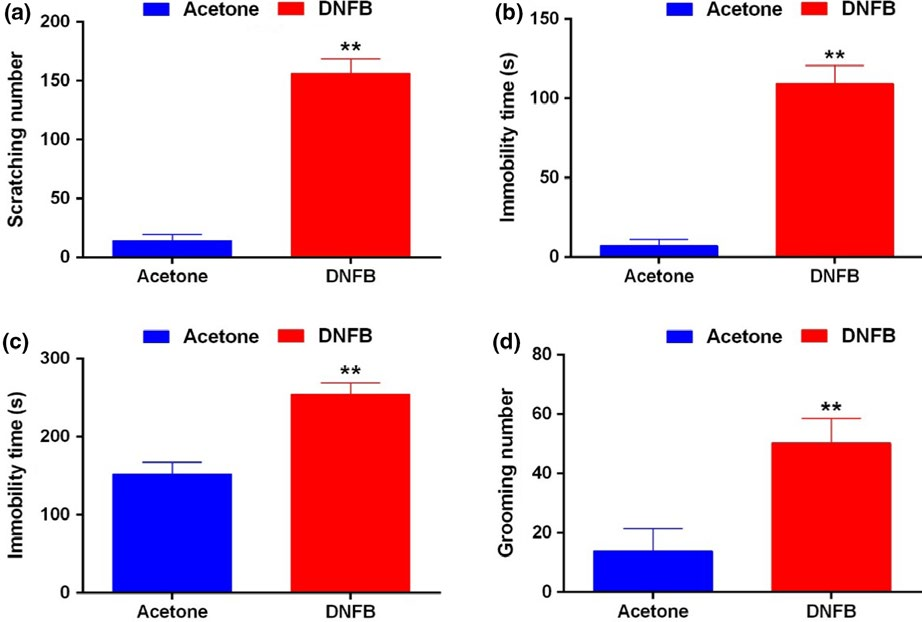
Chronic itch induced depressive behaviors. (a) The scratching number of the DNFB model mice was significantly increased compared to the control mice. (b–d) The immobility time in FST (b) and TST (c), and the number of grooming in ST (d) were all significantly increased in DNFB model than those in control mice. **p <* *.01 versus Control. FST, forced swimming test; TST, tail suspension test

The result of itch behavior showed that the scratch number of the DNFB model in 30 min was significantly higher than that of control group (*p *<* *.01, Figure 1a). The average scratch number of DNFB model mice reached 150 times in 30 min, suggesting that the DNFB model was successful. After scratch behavior recording, FST, TST, and ST tests were carried out to both DNFB model and control group. The results showed that the immobility time in FST and TST, and the frequency of grooming in ST were all significantly increased in the DNFB model group than that of control group (*p *<* *.01, Figure 1b–d). Rotarod test showed that compared to the control mice, the DNFB group exhibited no significant change of sustaining time on the rotarod (*p *>* *.05, Figure S1).

### 3.2 The establishment of chronic depression model and its scratching behaviors

**Figure 2:**
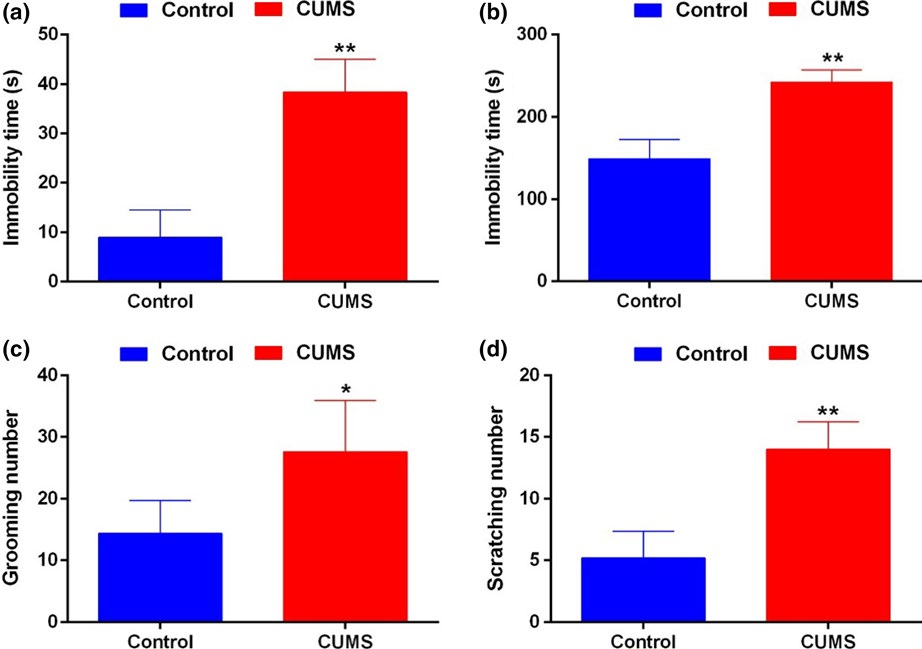
Chronic depression induced scratching behaviors. (a–c) The immobility time in FST (a) and TST (b), and the number of grooming in ST (c) were all significantly increased in the CUMS model than those of the control. (d) The spontaneous scratching number of the CUMS model significantly increased compared to that of control. *p <* *.05 versus Control; **p <* *.01 versus Control. CUMS, chronic unpredicted mild stress; FST, forced swimming test; TST, tail suspension test

After applying constant CUMS for 28 days, the immobility time of mice in FST, TST test were all significantly increased (*p* < .01, Figure 2a,b), and the frequency of grooming in ST was also increased (*p *<* *.05, Figure 2c). These behavior tests indicated that the CUMS model mice showed obvious depressive behavior. The spontaneous scratch number of CUMS model was also significantly increased compared to that of control (*p *<* *.05, Figure 2d).

### 3.3 Interaction deterioration between chronic itch and depression

**Figure 3:**
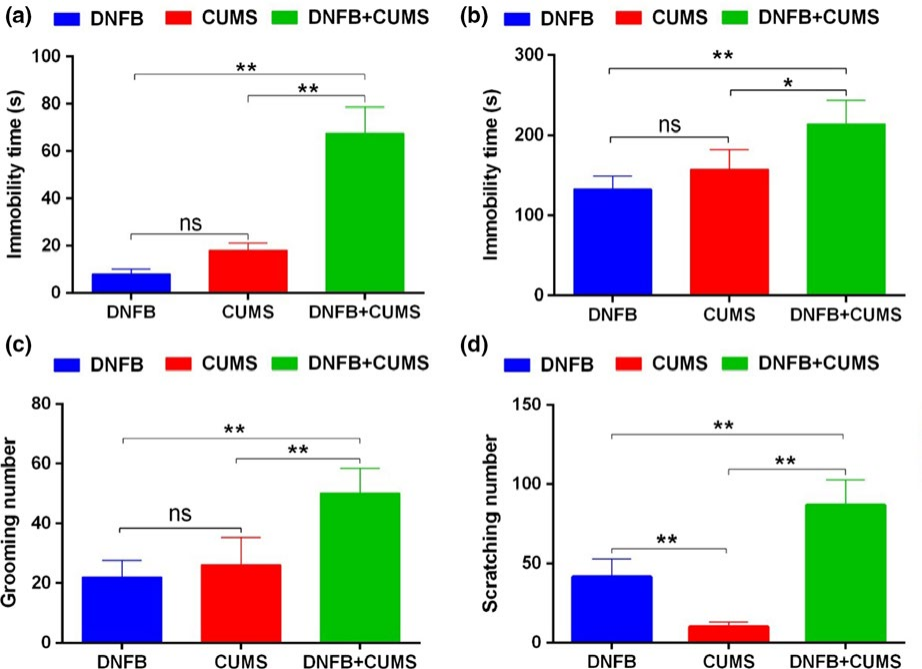
Chronic itch and depression are reciprocal causation and interaction deterioration. (a–c) Mice in group A (DNFB+CUMS) showed more depressive behaviors than those in group B (DNFB) and group C (CUMS) in the FST (a), TST (b) and ST (c) tests. (d) The scratch number in group A was significantly increased compared to that in group B and C. *p <* *.05 versus Group B; **p <* *.01 versus Group A or C. CUMS, chronic unpredicted mild stress; FST, forced swimming test

Mice were grouped according to the aforementioned requirements: group A were treated with both DNFB and CUMS for 14 days, while group B and group C only received DNFB and CMUS treatment, respectively. Fourteen days later, the results of FST, TST and ST tests indicated that mice in group A showed more obvious depressive behaviors than those in group B and C (*p *<* *.05 or *p *<* *.01, Figure 3a–c), which indicated the stimulation of chronic itch could accelerate the formation of depression. On the other hand, 14 days later, the scratch number of the mice in group A was significantly increased compared with that in group B and C, which indicated chronic depression could accelerate chronic itch formation (*p *<* *.01, Figure 3d).

### 3.4 Chronic depression promotes acute itch

**Figure 4:**
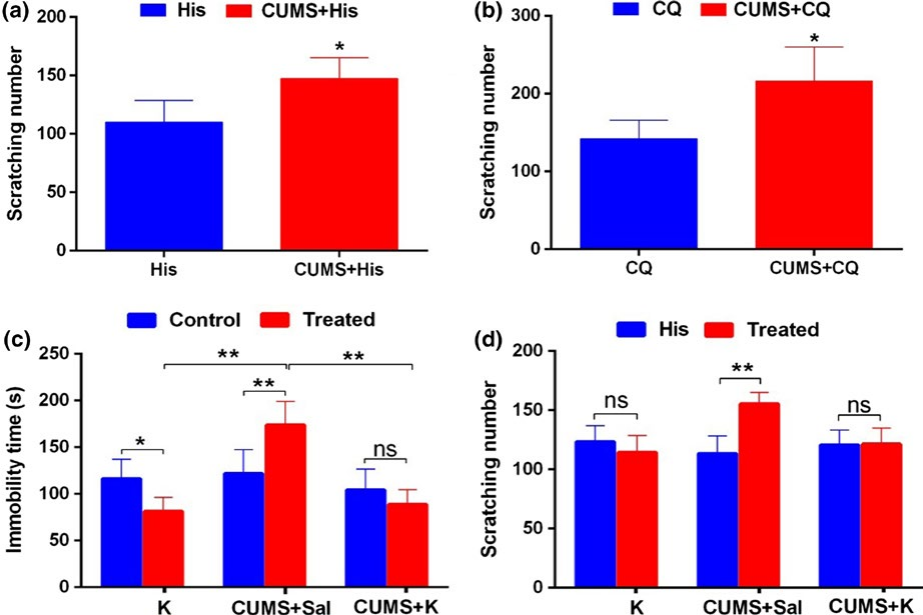
Chronic depression promotes acute itch. (a) Chronic depression promoted acute itch induced by histamine (His). (b) Chronic depression promoted acute itch‐induced by chloroquine (CQ). (c) Compared to the normal control, immobility time in FST test in CUMS+Saline mice and Ketamine mice were significantly decreased and increased, respectively, while the CUMS+Ketamine mice had no significant change. Compared to CUMS+Saline group, the Ketamine group and CUMS+Ketamine group exhibited less immobility time. (d) Compared to His‐control, only the CUMS+Saline mice expressed significantly increased scratching number. *p <* *.05; **p <* *.01. CUMS, chronic unpredicted mild stress; FST, forced swimming test; TST, tail suspension test

On the basis of the aforementioned CUMS model and the control mice, 20 μl histamine (His, 10 mg/ml) or chloroquine (CQ, 10 mg/ml) was intracutaneously injected into the back of neck. The scratching behavior test showed that scratching numbers of mice in CUMS+His group and CUMS+CQ group were all significantly increased compared with those of His‐injection group and CQ‐injection group, respectively (*p *<* *.05, Figure 4a,b).

After receiving twice (0th and 14th day) ketamine (a kind of anti‐depression drug) injection, mice showed less immobility time in the FST than that of normal control (*p *<* *.05, left columns of Figure 4c), which indicated that ketamine exerted anti‐depression effect in the mice. CUMS+Saline mice expressed obvious depression‐like behavior (middle columns of Figure 4c). The immobility time in the FST of CUMS+Ketamine mice had no obvious difference with that of normal control. Compared to A (Ketamine) and C (CUMS+Saline) groups, B (CUMS+Ketamine) group exhibited less immobility time in the FST, suggesting ketamine possesses potent anti‐depression effects (*p *<* *.01, Figure 4c). After His injection, the scratching number of CUMS+Saline mice was robustly increased comparing to that of control (*p *<* *.01, middle columns of Figure 4d), but no significant change was observed between group A and group B (*p *>* *.05, left and right columns of Figure 4d). These results showed that ketamine itself possessed no anti‐itch effect in normal mice, while after administration on CUMS mice, no depression like behavior was observed, and the increased His‐induced itch behavior also disappeared. All of these suggest that depression can facilitate His‐induced acute itch.

## 4 DISCUSSION

It is frequently observed in both clinical practice and real life that itch could cause negative emotions which could also induce or exacerbate itch sensation. Nevertheless, a systematic animal behavioral investigation is still lacking. In this present study, we established DNFB‐induced chronic pruritus model and CUMS‐induced chronic depression model, and systematically investigated the behavioral paradigm of interactive aggravation between itch and depression. The results will provide the behavioral basis for the study of related mechanisms.

In clinic, patients suffering from refractory pruritus dermatitis are often accompanied by neuropsychiatric diseases. Many patients diagnosed with chronic itch dermatitis, such as idiopathic dermatitis, neurodermatitis, psoriasis and systemic lupus erythematous, are afflicted by the “itch‐scratch‐itch” circle which will eventually lead to negative emotions such as anxiety and depression (van Laarhoven et al., 2012).

The prevailing theory for chronic itch‐induced depression is neuroendocrine hypothesis, which denotes that the occurrence of depression is deeply pertinent to the dysfunction of hypothalamus–pituitary–adrenal (HPA) axis (Catalan, Gallart, Castellanos, & Galard, 1998). In normal conditions, HPA axis could be activated by stress factors from both internal and external environments to confront the environmental changes, thus protecting our body from potential threats. However, as a source of chronic stress, the sustained “itch‐scratch‐itch” circle will lead to the dysfunction of HPA axis. Meanwhile, the sustained high‐level corticotrophin releasing hormone (CRH) within the cerebrospinal fluid will influence both the structure and the function of the limbic system, especially the hippocampus and amygdala nucleus which are most susceptible to injuries. All of these alterations may be related to the occurrence of depression (Rajkowska, 2003; Soares et al., 2013).

In previous studies concerning the negative emotions, people observed that CRH participate in the neurocircuits formed by cerebral cortex, thalamus, amygdala nucleus and bed nucleus of stria terminalis, mediate the regulation of the projections of GABAergic neurons and transform the sensory signals into anxiety and depression‐like behaviors. After injecting CRH into bilateral medial amygdaloidal nucleus, rats exhibited anxiety‐like behaviors that were reversed by CRH receptor 1 (CRHR1) antagonist antalarmin, indicating that CRH is directly involved in the formation of negative emotions via CRHR1 (Phelps & LeDoux, 2005; Vicentini, Cespedes, Nascimento, Bittencourt, & Viana, 2014). Another study suggested that the expression of brain derived neurotrophic factor (BDNF) and growth associated protein 43 (GAP‐43) in the hypothalamus was closely related to CRH in depression patients, and administration of CRH receptor antagonist could effectively downregulate the content of BDNF and GAP‐43, and reversed the depression‐like behaviors of animals (Dai, Zhang, & Chen, 2015). To sum up, the sustained high‐level of CRH under stress condition may cause depression by regulating the expression of its downstream proteins.

Another hypothesis for itch‐induced depression is the dermal inflammation hypothesis. It is well‐known that both the skin and the nervous system derive from ectoderm so that they may share some neurokines and receptors (Drolet, Prendiville, Golden, Enjolras, & Esterly, 1995), which may be the foundation for the comorbidity of skin diseases and neuropsychiatric disorders. Especially, in the condition of pruritus dermatitis, the released inflammatory factors will influence the function of central nervous system. Although the inflammatory factors such as TNF‐α and IL‐1β are produced in peripheral inflammation areas where they could not pass through blood brain barrier, they could get access to the central nervous system via some unique mechanisms (Banks, 2005; Goehler et al., 2000; Turrin & Rivest, 2004) and contribute to the synthesis of cytokines in central nervous system, thus activating hypothalamus and other brain regions to participate in the pathogenesis of depression. This hypothesis provides valuable clues for our study about the relationship between chronic pruritus dermatitis and depression.

For the neuroendocrine and inflammatory hypothesis of the aforementioned interaction of itch and negative emotions, the present study cannot prove which one plays a leading role. On one hand, the CUMS model can induce the increase of inflammatory cytokines as well as hormones (Liu et al., 2017). On the other hand, the DNFB chronic itch model is mainly caused by the repeated application of DNFB to nape skin leading to atopic dermatitis‐like pathological alterations, which is closely related to inflammation mechanisms (Sasso et al., 2017). Therefore, the interaction between itch and depression may involve both hypotheses.

Nowadays, various conflicts and competitions in the society impose a major impact on people's mental health, along which is the high morbidity of depression. Therefore, it is urgent to study the pathogenesis of depression (Phillips et al., 2002). It is observed in clinical practice that apart from typical syndromes of depression, many depressive patients exhibited the symptoms of pruritus. Many patients initially diagnosed with pruritus were finally diagnosed with depression, and obtained obvious relief after antidepressant drugs treatment. In the present study, we also observed that the anti‐depression drug ketamine (Abdallah et al., 2016; Brachman et al., 2016) could significantly inhibit the depression‐like behaviors of CUMS mice, and simultaneously stopped the promoting effect on His‐induced acute itch. All of these indicated that depression could induce or aggravate itch sensation. There has been several studies concerning chronic stress (depression)‐induced itch hypersensitivity. Amano et al. (2008) observed that 4 weeks after WAS treatment, atopic dermatitis could be induced under sterile conditions in Nc/Nga mice, which could be completely blocked by pretreatment of CRH. Zhao et al. (2013) found that WAS‐evoked chronic stress mice models were more sensitive to compound 48/80. In addition, the number of mastocytes within skin and the serum level after pruritogen injection were significantly increased compared to those of control mice, indicating that chronic stress could induce or facilitate pruritus. Peng et al. (2015) observed that heterotypic chronic intermittent stress (HIS) could significantly enhance 5‐HT‐induced scratching behaviors in rats, which may be mediated by the up‐regulation of proinflammatory factors via β2‐adrenoceptors (β2‐AR).

Skin is a “speakable” organ that directly reflects our mental activities, given that our face becomes flushed when we are excited and become pale when we are terrified. Some depression patients often exhibit spontaneous itch in scalp or body skin, and some cases are possibly related to the link between nervous system and immune system. Moreover, in this neuro‐immune link, the role of the HPA axis and its key mediator CRH are gaining more and more attention (Nejati, Kovacic, & Slominski, 2013). Clinical studies indicated that the increased level of CRH within cerebrospinal fluid (CSF) in depression patients was in accordance with the increased number of CRH‐positive neurons in the paraventricular nucleus of hypothalamus (Merali et al., 2004). A previous study concerning the relationship between depression and pain indicated that a lower dose of CRH injection into rat central amygdaloid nucleus (CeA) could induce the hyperalgesia via CRHR1, which may contribute to explain the “migratory pain” in depression patients. In light of this, CRH may possibly be involved in the progress of the interactive aggravation of depression and itch (Cui, Lundeberg, & Yu, 2004; Ji & Neugebauer, 2008). Some researches showed that increased CRH in chronic stress or depression patients could directly bind to CRHR1 expressed on skin mastocytes and induce degranulation (Arck & Paus, 2006; Chrousos, 1995; Theoharides et al., 1998). Once activated, mastocytes release a large amount of itch mediators, such as histamine, substance P, protease, leukotrienes and prostaglandin, and then induce itch sensation (Arck & Paus, 2006; Chrousos, 1995; Paus, Theoharides, & Arck, 2006; Theoharides et al., 1998).

In fact, in clinical practice, it is difficult to differentiate the causal relationship between pruritus and depression since in most cases these two diseases show high comorbidity. In this study, we exquisitely displayed the interactive aggravation of pruritus and depression via behavioral methods, which will pave the way for subsequent studies concerning the detailed mechanisms. Intriguingly, stress is the common character of depression and pruritus. Once an adverse event such as pruritus occurs, the HPA axis will be activated to release CRH. Sustaining CRH release has been demonstrated to induce both the occurrence and the interactive aggravation of depression and pruritus, and thus forming a vicious circle which worsens the condition. It is urgently needed to search for a point where this vicious cycle could be interrupted, which will provide potential drug targets for the efficacious treatment of depression and pruritus.

## 5 CONCLUSION

We exquisitely demonstrated that there is cross aggravation effect between pruritus and depression by using chronic itch model, chronic depression model and chronic itch‐depression model in mice. These cross aggravation experimental models will provide behavioral basis for the study of related mechanisms.

## CONFLICT OF INTEREST

The authors declare that they have no conflict of interest.
