# Supplementary figures and images for: The behavioral study on the interactive aggravation between pruritus and depression
Source: Brain Behav. 2018 May 1;8(6):e00964. doi: 10.1002/brb3.964 (PMC5991569; doi:10.1002/brb3.964)

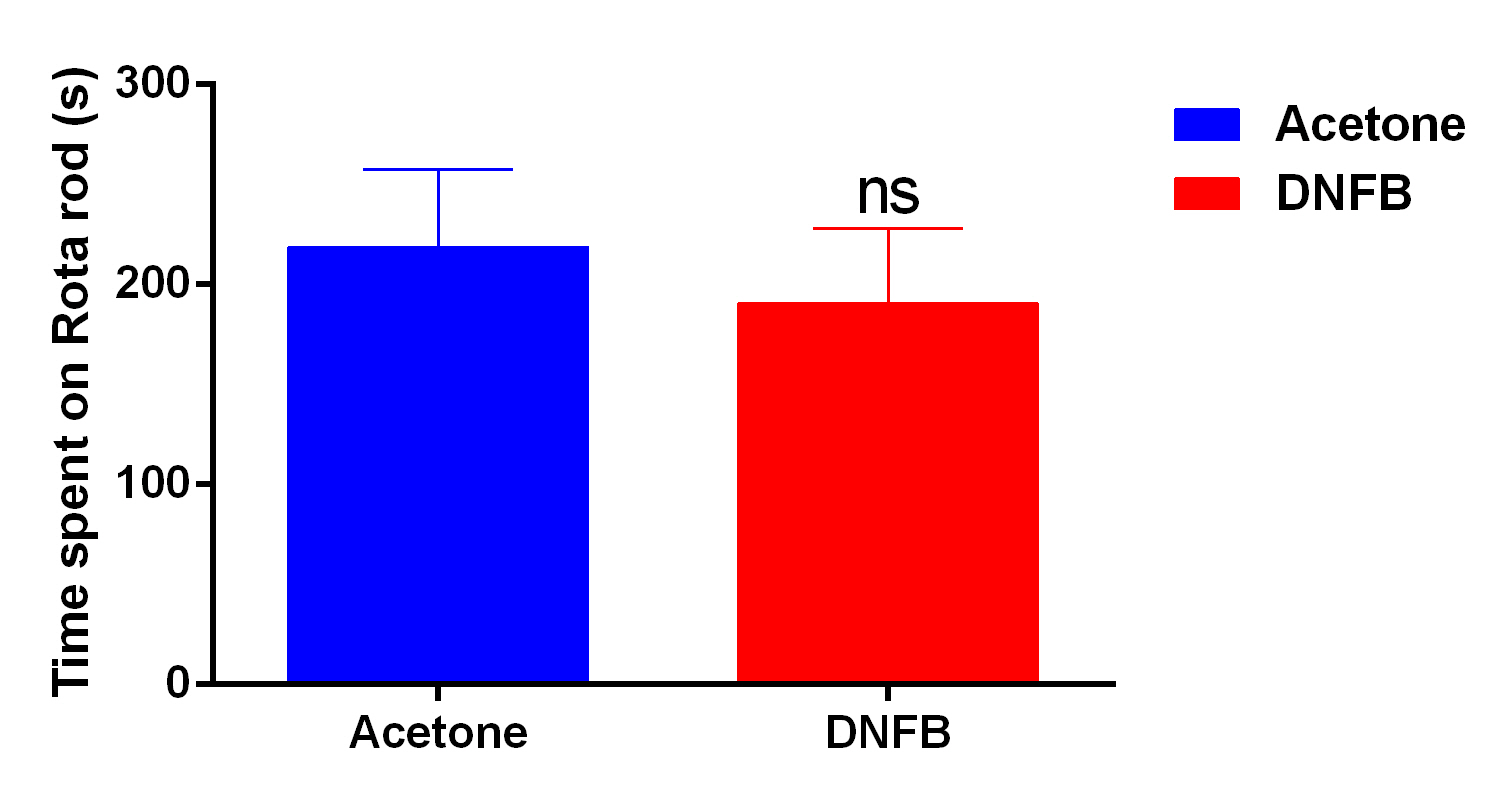

Supplement: Supplementary file 1 [file BRB3-8-e00964-s001.jpg]
